# Analysis on the Susceptibility Genes in Two Chinese Pedigrees with Familial Parkinson's Disease

**DOI:** 10.1155/2010/674740

**Published:** 2010-08-23

**Authors:** Changshui Xu, Jun Xu, Yanmin Zhang, Jianjun Ma, Hideshi Kawakami, Hirofumi Maruyama, Masaki Kamada

**Affiliations:** ^1^Department of Neurology, Henan Provincial People's Hospital, Zhengzhou 450003, China; ^2^Department of Epidemiology, Research Institute for Radiation Biology and Medicine, Hiroshima University, Hiroshima 734-8553, Japan

## Abstract

*Objective*. To screen the susceptibility genes in Chinese pedigrees with early-onset familial Parkinson's disease (FPD). *Methods*. Fifty-one genomic DNA samples extracted from two Chinese pedigrees with FPD, the alpha-synuclein genes (SNCA), the leucine-rich repeat kinase 2(LRRK2), PINK1(PTEN-induced putative kinase 1), PARK7(Protein DJ1), PARK2(Parkinson juvenile disease protein 2), the glucocerebrosidase (*GBA*), and ATP(Ezrin-binding protein PACE-1), were sequenced by the use of polymerase chain reaction (PCR) technique. The gene dose of SNCA was checked. *Results*. There were only two missense mutations observed, respectively, at exon 5 of LRRK2 and exon 10 of PARK2, and both were enrolled in SNPs. *Conclusion*. No meaningful mutations could be detected, and other susceptibility genes should be detected in FDP patients in China.

## 1. Background

Parkinson's disease (PD) is characterized by the degeneration of dopaminergic neurons in the substantia nigra in midbrain. The etiology of PD is multifactorial, and PD is likely to involve different factors in different genetically related populations. Several different genes have been identified as susceptibility genes of familial PD(FDP)including alpha-synuclein gene (SNCA) and the leucine-rich repeat kinase 2 (LRRK2) mutations, which underlie autosomal dominant forms of PD, and mutations or multiplications of PINK1(PTEN-induced putative kinase 1), PARK7(Protein DJ1), and PARK2(Parkinson juvenile disease protein 2), with autosomal recessive forms of PD [1–4], especially in Chinese [5, 6]. Recent studies found an increased frequency of mutations in the gene encoding glucocerebrosidase (*GBA*), a deficiency of which causes Gaucher's disease in patients with Parkinson's disease [7]. ATP(Ezrin-binding protein PACE-1) variant is related to FPD too [8, 9]. We selected 51 genomic DNA samples from two Chinese pedigrees with FPD to screen the SNCA, LRRK2, PINK1, DJ1, parkin2, *GBA,* and ATP genes. 

## 2. Subjects and Methods

We selected two Chinese pedigrees with typical FPD from two families. Family 1 was from outpatient department of neurology, including 4 PD patients and 17 related individuals, and family 2 was from a disease census survey in rural areas, including 4 PD patients and 26 related individuals. All PD patients were diagnosed based on UK PD Brain Bank Clinical diagnostic criteria [10] and were followed up for over 3 years.

Fifty-one genomic DNA samples were from two Chinese pedigrees with FPD. A total of 106 pairs of primers were designed in coding region of the alpha-synuclein genes (SNCA, chr4: 90647204-90758290), the leucine-rich repeat kinase 2(LRRK2, chr12: 40618813-40763075), PINK1(chr1: 20959948-20978004), PARK7(DJ1: chr1: 8021752-8045565), PARK2(chr6: 161768452-163148803), the glucocerebrosidase (*GBA,chr1: 155204243-155211025), *and the ATP(Ezrin-binding protein PACE-1, chr1: 169821804-169863076). Polymerase chain reaction (PCR) was carried, and the PCR products were sequenced with 3130xl genetic sequence analyzer (Applied Biosystems). The gene dose of SNCA was checked by ABI 7900HT Fast Real-Time PCR system (Applied Biosystems) using THUNDER BIRD SYBR qPCR Mix (TOYOBO, Japan) (Table 1).

## 3. Results

**Figure 1:**
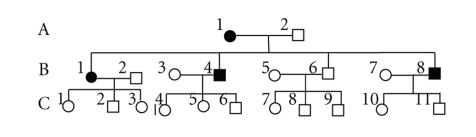
(family 1).

**Figure 2:**
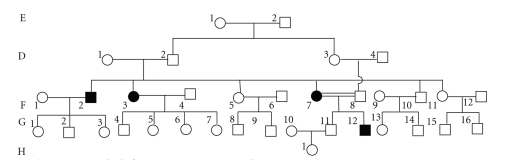
(family 2).

The characteristics of the studied Chinese pedigrees are presented in following Figures 1 and 2.

Family 1 included 4 PD patients. A1 manifested only rest tremors at 58 years old, followed by muscular rigidity, and the diagnosis was made at the age of 62 years old when postural instability appeared. The early onset of rest tremors in B1, B4, and B8 occurred at 24 years old, followed by postural instability, and the diagnosis was made at the age of 27 years old when muscular rigidity appeared. Family 2 included 4 PD patients. F2, F3, and F7 manifested rest tremors at 29 years old, followed by postural instability, and the diagnosis was made at the age of 32 years old when muscular rigidity appeared. G12 manifested rest tremors at as early as 16 years old, postural instability, and muscular rigidity, respectively, at 17 years old, and the symptoms were very serious. All above patients were sensitive to L-dopa and anticholinesterase medicines, but G12 sensitivity was gradually lowered, and the therapeutic effect was not good.

 Only one member (H1, female, 9 years old) was detected to have synonymous mutation at exon 5 of LRRK2 from the two Chinese pedigrees with FPD, rs10878245, c.578 T > C. One member (the early onset was at 17 years old) was observed to have a missense mutation at exon 10 of PARK2, rs1801582, c.1272 G > C (p.380 V > L). No mutation was found by sequencing the other exons. There was no *duplication or deletion of SNCA.*

## 4. Discussion

 We have screened the susceptibility genes causing FDP in China, but have not found any meaningful mutations. These observations suggest that the reported mutations are not the predominant genotype of Chinese FPD. The mutations of patients with FPD in the mainland China may be different from those in Europe, America, and other different genetically related populations. They are of ethnic and geographical distribution [11]. 

 F row patients are attacked at 32 years of age, but G12 patient at as early as 17 years old, and the clinical symptoms are serious due to intermarriage. By guessing, the inheritance maps of family 2, G12 has homozygous state of causative genes of PD. Doubling of the mutant gene may accelerate the onset of PD. 

We have investigated only 8 FPD patients, which could not represent the entire Chinese PD. A, B, and C rows of family 1 live in the same environment and are on similar diet. D, E, F, G, and H rows of family 2 are the same too. The age range of C1 to C11 of family 1 is from 3 to 23 years old, and of G and H rows of family 2 are from 9 to 30 years old, who could be too young to exhibit PD clinical symptoms except for G12 due to intermarriage. The number of potential PD patients is probably larger than 20 cases in the two Chinese pedigrees. The genotype association is consistent relatively because two Chinese pedigrees are of one pedigrees' FPD, and G12 may be in homozygous gene state, so the positive rate of the susceptibility genes should be high. We conclude that multifactorial and genetic polymorphic maybe the genuine etiology of PD [1, 9], but not dependent genotype. Perhaps, impaired mitochondrial dynamics and function are responsibile for Chinese FPD [12–14], or defects in repairing oxidative DNA damage can lead to a number of neurological disorders, as Alzheimer and Parkinson's disease [15]. The above genes duplication or triplication probably induces mutations of PD [16, 17], and we are going to do it for Chinese FPD in the next step. 

## 5. Additional Information

**UK Parkinson's Disease Society Brain Bank Clinical Diagnostic Criteria**

**Step 1 (diagnosis of Parkinsonian syndrome)**

- Bradykinesia.
- At least one of the following:
  - muscular rigidity,
  - 4–6 Hz rest tremor,
  - Postural instability not caused by primary visual, vestibular, cerebellar, or proprioceptive dysfunction.

**Step 2 (exclusion criteria for Parkinson's disease)**

- History of repeated strokes with stepwise progression of parkinsonian features.
- History of repeated head injury.
- History of definite encephalitis.
- Oculogyric crises.
- Neuroleptic treatment at onset of symptoms.
- More than one affected relative.
- Sustained remission.
- Strictly unilateral features after 3 years.
- Supranuclear gaze palsy.
- Cerebellar signs.
- Early severe autonomic involvement.
- Early severe dementia with disturbances of memory, language, and praxis.
- Babinski sign.
- Presence of cerebral tumor or communication hydrocephalus on imaging study.
- Negative response to large doses of levodopa in absence of malabsorption.
- MPTP exposure.

**Step 3 (supportive prospective positive criteria for Parkinson's disease)**

Three or more required for diagnosis of definite Parkinson's disease in combination with step one.

- Unilateral onset.
- Rest tremor present.
- Progressive disorder.
- Persistent asymmetry affecting side of onset most.
- Excellent response (70%–100%) to levodopa.
- Severe levodopa-induced chorea.
- Levodopa response for 5 years or more.
- Clinical course of ten years or more.

## Figures and Tables

**Table 1:** 

1. PCR			
DW	15.1 *μ* L	14.1 *μ* L	
DMSO	—	1.0 *μ* L	94°C 10 min–94°C 30 sec↓–72°C 10 min–4°C
10 × PCR Buf	2.0 *μ* L	2.0 *μ* L	52°C to 60°C 30 sec↓
2.5 mM dNTP	1.0 *μ* L	1.0 *μ* L	72°C 45 sec↓
3.5 U/ul HiFi	0.3 *μ* L	0.3 *μ* L	×40 cycles
10 pm For primer	0.5 *μ* L	0.5 *μ* L	
10 pm Rev primer	0.5 *μ* L	0.5 *μ* L	
DNA	0.6 *μ* L	0.6 *μ* L	

Total	20.0 *μ* L	20.0 *μ* L	

2. Sequence			
DW	6.3 *μ* L		
1 × Seq Buf	1.0 *μ* L		96°C 1 min–96°C 10 sec↓–4°C
BigDye	0.2 *μ* L		60°C 4 min↓
0.8 pm For primer (or Rev primer)	2.0 *μ* L		×30 cycles
Template DNA	0.5 *μ* L		

Total	10.0 *μ* L		

3. Denature			95°C 5 min–4°C

4. QPCR			
THUNDERBIRD SYBR qPCR Mix	9 *μ* L		95°C 1 min–95°C 15 sec
Forward Primer	6 pmol		57°C 20 sec
Reverse Primer	6 pmol		72°C 40 sec
50× ROX reference dye	0.36 *μ* L		×40 cycles
Template DNA	1 *μ* L		
	Upto18 microlitter		
